# Effects of Dietary 1,8-Cineole Supplementation on Growth Performance, Antioxidant Capacity, Immunity, and Intestine Health of Broilers

**DOI:** 10.3390/ani12182415

**Published:** 2022-09-14

**Authors:** Yuting Di, Aizhi Cao, Yuxin Zhang, Juntao Li, Yongbo Sun, Shixia Geng, Yongchen Li, Liying Zhang

**Affiliations:** State Key Laboratory of Animal Nutrition, Ministry of Agriculture and Rural Affairs Feed Industry Centre, China Agricultural University, Beijing 100193, China

**Keywords:** 1,8-cineole, broiler, growth performance, antioxidant capacity, immunity, intestinal health

## Abstract

**Simple Summary:**

1,8-cineole is a cyclic monoterpene compound, which can be extracted from plants or synthesized artificially. Dietary supplementation with plant essential oil containing 1,8-cineole can improve the performance of broiler chickens. However, limited literature exists on the supplementation of purified 1,8-cineole in diets for broiler chickens under normal conditions without stress challenging. Therefore, the purpose of this study was to evaluate the effects of purified 1,8-cineole on growth performance, antioxidant capacity, immunity, and intestinal health of broilers and its optimal dose in the diets. The results showed dietary supplementation of 1,8-cineole had positive effects on broilers.

**Abstract:**

This study was conducted to investigate the effects of 1,8-cineole on antioxidant capacity, immunity, and intestinal health of broilers. A total of 540 1-day-old Arbor Acres (AA) male broilers were randomly divided into five treatments with six replicates per treatment, and 18 broilers per replicate for 42 days. Dietary treatments were a corn–soybean meal basal diet supplemented with 0, and 10, 20, 30, and 40 mg/kg 1,8-cineole, respectively. Dietary supplementation with 20~30 mg/kg of 1,8-cineole increased the ADG from d 22 to 42 and d 1 to 42 (*p* < 0.05), and decreased the FCR (*p* < 0.05). Dietary supplementation of 10~40 mg/kg of 1,8-cineole increased total antioxidant capacity (TAOC) in serum (*p* < 0.05), and decreased malondialdehyde (MDA) level in the liver on day 21 (*p* < 0.05). The supplementation of 20~30 mg/kg of 1,8-cineole increased the activity of total superoxide dismutase (T-SOD) in the serum and liver and TAOC in the serum and the liver (*p* < 0.05), and decreased the level of MDA in the serum and the liver (*p* < 0.05) on day 42. Dietary supplementation with 20~30 mg/kg of 1,8-cineole increased serum immunoglobulin A, immunoglobulin G, and immunoglobulin M contents on day 21 (*p* < 0.05). On day 21, dietary supplementation of 20~30 mg/kg of 1,8-cineole increased the VH and VH/CD (*p* < 0.05) in the jejunum and ileum. The supplementation of 20~30 mg/kg of 1,8-cineole increased the content of secretory immunoglobulin A in the duodenum and ileum mucosa on d 42 (*p* < 0.05). In conclusion, dietary supplementation of 1,8-cineole improves the growth performance of broilers by enhancing antioxidant capacity, immunity, and intestinal morphology.

## 1. Introduction

**Scheme 1:**
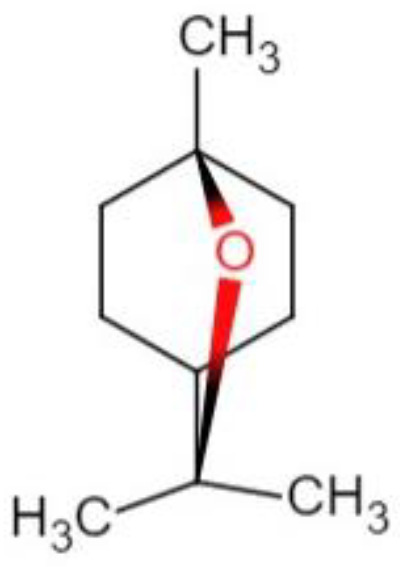
The chemical structural formula of 1,8-cineole.

1,8-cineole is a cyclic monoterpene compound, which is also known as eucalyptol. Its chemical name is 1,3,3-trimethyl-2-oxabecyclic [2,2,2] octane and the chemical structural formula is shown in Scheme 1. 1,8-cineole can be extracted from Thyme [1], *Alpiniae zerumbet* [2], and *Eucalyptus* [3] or synthesized chemically [4]. Besides 1,8-cineole, the crude essential oils obtained by extraction contain numerous monoterpenes, oxymonoterpenes, sesquiterpenes, and oxysesquiterpenes [5]. 1,8-cineole owns antibacterial [6], anti-inflammatory [7], and antioxidant [8] activities. 1,8-cineole is widely applied in the prevention and treatment of respiratory [9] and cardiovascular [10] diseases.

Studies have reported the application of plant essential oils containing 1,8-cineole in animal production, especially in broiler production. Providing essential oil, a mixture of *Eucalyptus* and peppermint, by drinking water to broiler chickens, improved the growth performance under the challenges of Newcastle disease virus [11] or *Eimeria* spp. [12]. Under heat stress conditions, Tekce, et al. [13] also found a blend of thyme, *Eucalyptus*, and clove oils had a positive effect on growth performance. The addition of blended thyme, peppermint, and *Eucalyptus* essential oils to drinking water improved performance of broilers even under non-stress conditions [14]. Otherwise, Mustafa [15] found that dietary supplementation of *Eucalyptus* essential oil could improve growth rate and feed conversation under cold stress conditions. *Eucalyptus* essential oil enhanced the antioxidant capacity and increase the apparent ileal digestibility of nutrients in broiler chickens [16]. However, plant essential oils contain not only 1,8-cineole, but also other ingredients. The literatures rarely relates to the effect of purified 1,8-cineole in broiler chickens under no challenged conditions, and the optimal inclusion level of 1,8-cineole in diets of broiler chickens is uncertain. Therefore, the purpose of this study was to evaluate the effects of 1,8-cineole on growth performance, antioxidant capacity, immunity, and intestinal health of broilers and determine the optimal inclusion level in the diets.

## 2. Materials and Methods

The experimental procedures were approved by the China Agricultural University Animal Care and Use Committee (Beijing, China; NO. AW91702202-1-10; 19 July 2022). This study was performed in the Fengning Research Unit of China Agricultural University (Hebei, China).

### 2.1. 1,8-Cineole

1,8-cineole was provided by Shandong Longchang Animal Health Products Co., Ltd. (Dezhou, China), which is extracted from *Eucalyptus polybractea*. The purity of 1,8-cineole was 91.5% which was determined by gas chromatography according to the method specified in the product standard.

### 2.2. Experimental Broiler Chickens, Design, Management, and Diets

A total of 540 1-day-old Arbor Acres (AA) broiler chickens were purchased from Beijing Arbor Acres Poultry Breeding Co., LTD. All broiler chickens were randomly assigned to five dietary treatments, with 18 per cage and 6 replicate cages per treatment. All broilers were raised in wire-floored cages (0.9 × 0.6 × 0.4 m) which were equipped with 2 nipple drinkers and a feeder in an environmentally controlled room. The control group was fed a corn–soybean meal basal diet, and the other four groups were fed the basal diet supplemented with 10, 20, 30, and 40 mg/kg 1,8-cineole, respectively. The experiment lasted for 42 days.

Nutrient compositions of the corn–soybean meal basal diet satisfied the nutritional requirements of broilers (NRC, 1994) [17]. The compositions and nutrient content of the basal diet are shown in Table 1. During the formulation of the diets, 1,8-cineole was firstly diluted 1000 times with soybean oil by a step-by-step dilution before mixed with other ingredients. Birds were fed the starter diet from day 1 to day 21 and the grower diets from day 22 to day 42 in mash form, and had free access to feed and water. The broiler chickens were provided with 24 h of light during the first 3 days, and with a 23 h of light in subsequent days until day 42. Temperature was controlled at 34~35 °C during the first week and gradually decreased by 2 °C every week. The final temperature was kept at 22~24 °C, and the relative humidity was 45~55%. All broiler chickens were inoculated with Newcastle disease and bronchial vaccine by eye-drop and nose-drop on day 7 and vaccinated with bursal disease vaccine or Newcastle disease vaccine by drinking water on d 14 and day 21.

### 2.3. Growth Performance

On day 1, initial weight of broiler chicken was weighed individually. Body weight and feed intake were recorded in each replicate and broiler chickens were weighed after 12-h fasting on day 21 and day 42. And average daily gain (ADG), average daily feed intake (ADFI), and feed to gain ratio (FCR) were calculated for days 1–21, days 22–42, and days 1–42.

### 2.4. Sample Collections

On day 21 and day 42, one broiler chicken whose body weight was close to the average body weight of each pen was selected for blood collection (*n* = 6) from the wing vein. Blood samples were placed at room temperature for 30 min, and centrifuged at 3000 g for 15 min for serum preparation before storage at −20 °C for later analysis. After blood collection, birds were euthanasially killed. Tissues of liver, mucosa of the duodenum, jejunum, and ileum, and tissue in the mid of the jejunum and ileum were collected immediately. All samples were placed in liquid nitrogen immediately for quick freezing and then stored at −20 °C for further analysis.

On day 21 and day 42, one broiler chicken with close to average body weight was slaughtered in each replicate. Jejunum and ileum of 2 cm sample were separated, flushed with 0.9% physiological saline solution, fixed with 4% paraformaldehyde solution, and stored at 4 °C. The intestinal tissue was fixed and paraffin sections were made. It was stained with hematoxylin and eosin and observed under a light microscope. To measure the morphological parameters of intestinal structure, 6 well-oriented sections were randomly selected from each group for observation, and then the villus height (VH) and crypt depth (CD) of each section were measured and recorded in the randomly selected microscopic field using Image-Pro Plus 6.0 software (Media Cybernetics, Inc., Silver Spring, MD, USA). The ratio of villus height to crypt depth was calculated.

### 2.5. Sample Analysis

Association of Official Analytical Chemists [18] were used when testing nutrients in the diets including total phosphorus (AOAC method 995.11), calcium (AOAC method 927.02), dry matter (DM) (AOAC method 934.01), crude protein (CP) (AOAC method 988.05), and amino acids (AOAC method 994.12). Before acid hydrolysis by 6 M HCl, performic acid oxidation was administrated to determine dietary methionine determination.

Glutathione peroxidase (GSH-Px) and superoxide dismutase (T-SOD) activities, total antioxidant capacity (TAOC), and malondialdehyde (MDA) contents in serum and liver were determined according to the instructions of the kit of Nanjing Jiancheng Institute Bioengineering Institute (Nanjing, China). The levels of immunoglobulin A (IgA), immunoglobulin M (IgM), and immunoglobulin G (IgG) in serum were determined with commercial kits of Beijing Zhongshan Boao Biological Technology Co., LTD (Beijing, China). The levels of tumor necrosis factor-α (TNF-α), interleukin-1 β (IL-1β), interleukin (IL-4), and interleukin (IL-10) in the serum, and the content of SIgA in the duodenum, jejunum, and ileum mucosa were measured with a commercial kit of Beijing Kangjia Hongyuan Biotechnology Co., LTD (Beijing, China).

### 2.6. Statistical Analysis

Data of this study were analyzed by one-way analysis of variance with the GLM procedure of SAS 9.4 (SAS Institute Inc., Cary, NC, USA). The pen was used as an experimental unit for growth performance, while individual bird per cage was used as experimental unit for analysis of antioxidant capacity, immunity, intestinal morphology, and intestinal mucosa SIgA. Duncan’s multiple comparison test was used to determine the difference among treatments. The linear and quadratic effects of 1,8-cineole levels were evaluated using orthogonal polynomial contrasts. A quadratic regression fitting curve model was performed using GraphPad Prism 9 to evaluate the optimal level of 1,8-cineole in the diets. Difference was deemed significant when the *p*-value was less than 0.05.

## 3. Results

### 3.1. Growth Performance

As shown in Table 2, dietary 1,8-cineole inclusion increased ADG (quadratic, *p* < 0.05), but did not affect ADFI and FCR (*p* > 0.05) during d 1~21. ADG increased (linear and quadratic, *p* < 0.05), and FCR decreased (quadratic, *p* < 0.05) with the increase in dietary 1,8-cineole supplementation level during days 22~42 and days 1~42. Furthermore, ADFI was increased (linear, *p* < 0.05) during days 22~42. According to the quadratic fit model of dietary 1,8-cineole supplementation level, ADG (Y = −4.150 × 10^−3^X^2^ + 0.2199X + 50.84, R^2^ = 0.78) and FCR from (Y = 9.286 × 10^−5^X^2^ − 4.164 × 10^−3^X + 1.735, R^2^ = 0.88) from days 1~42, the optimal supplemental level of 1,8-cineole for broiler chickens was 26.49 mg/kg and 24.84 mg/kg, respectively, and the optimal dose range was 18.45–34.54 mg/kg and 20.59–29.09 mg/kg, respectively.

### 3.2. Antioxidant Capacity

Dietary 1,8-cineole inclusion increased T-SOD activity (quadratic, *p* < 0.05, Table 3 and Table 4) in the liver, TAOC (quadratic, *p* < 0.05) in serum and liver, and decreased the level of MDA (linear and quadratic, *p* < 0.05) in the liver on day 21, but there was no effect on GSH-PX activity (*p* > 0.05) in the serum and liver. As dietary 1,8-cineole supplementation level increased, T-SOD activity and TAOC level increased (linear and quadratic, *p* < 0.05) in serum and liver, while MDA level in the serum and liver decreased (linear and quadratic, *p* < 0.05) on day 42.

### 3.3. Immune Parameters

As shown in Table 5, the concentration of IgA, IgG, and IgM in the serum showed a linear and quadratic response (*p* < 0.05) to the increase in dietary 1,8-cineole supplementation level on day 21. However, there were no significant differences in the concentrations of IgA, IgG, and IgM among all groups on day 42 (*p* > 0.05).

The level of TNF-α in the serum was linearly decreased (*p* < 0.05, Table 6), while the level of IL-10 and IL-4 was quadratically increased (*p* < 0.05) on day 21 with increasing levels of 1,8-cineole. Dietary 1,8-cineole inclusion decreased the level of TNF-α and IL-1β (linear and quadratic, *p* < 0.05) in the serum, but increased the level of IL-10 (quadratic, *p* < 0.05) on day 42.

### 3.4. Intestinal Morphology

**Figure 1:**
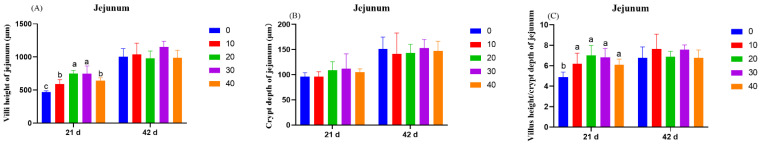
Effects of dietary 1,8-cineole supplementation on jejunum morphology of broiler chickens: (**A**) villi height; (**B**) crypt depth; (**C**) villi height/crypt depth. ^a,b,c^ Means within a row with different superscripts are significantly different (*p* < 0.05).

**Figure 2:**
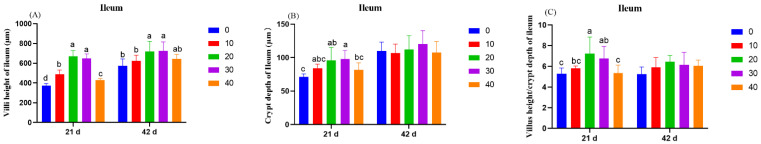
Effects of dietary 1,8-cineole supplementation on ileum morphology of broiler chickens: (**A**) villi height; (**B**) crypt depth; (**C**) villi height/crypt depth. ^a,b,c,d^ Means within a row with different superscripts are significantly different (*p* < 0.05).

As shown in Figure 1 and Figure 2 on day 21, dietary supplementation of 20~30 mg/kg of 1,8-cineole increased the VH and VH/CD (*p* < 0.05) in the jejunum and ileum. On day 42, the addition of 20~30 mg/kg of 1,8-cineole increased the VH (*p* < 0.05) in the ileum, while the VH, CD, and VH/CD of jejunum were not affected (*p* > 0.05).

### 3.5. Intestinal Mucosa SIgA

As shown in Table 7, the contents of SIgA in the mucosa of the duodenum, jejunum, and ileum linearly increased (*p* < 0.05) in broiler chickens fed diets with 0~40 mg/kg 1,8-cineole on day 21. Dietary 1,8-cineole supplementation increased the content of SIgA in duodenal mucosa (quadratic, *p* < 0.05) and in ileal mucosa (linear and quadratic, *p* < 0.05), while the content of SIgA in the jejunum was not affected by 1,8-cineole level (*p* > 0.05).

## 4. Discussion

Reported studies have showed that *Eucalyptus* essential oil with 1,8-cineole could improve the growth performance of broiler chickens. For example, dietary supplementation with 500~2000 mg/kg of *Eucalyptus* essential oil that contains 35.17% 1,8-cineole increased the ADG of Ross 308 broilers under cold stress conditions [15]. Mohebodini et al. [16] reported that dietary supplementation of 1000 mg/kg *Eucalyptus* essential oil with 67.85% 1,8-cineole improved ADG and the ratio of gain to feed of Ross 308 broilers. This study also found that dietary supplementation of 1,8-cineole improved the ADG and ADFI, and decreased the FCR. And the optimal inclusion level of 1,8-cineole was 20~30 mg/kg. The positive effect of 1,8-cineole on the growth performance of broilers may be attributed to the improvement of intestinal morphology [14], the stimulation of amylase, lipase, and trypsin secretion [15], and the enhancement of immune response [19]. In addition, according to the results of this study, the improvement in the growth performance of broilers may be related to the improvement in the intestinal mucosal immunity.

Antioxidant enzymes such as SOD, CAT, and GSH-PX play a major role in enhancing the antioxidant defense of cells against oxidative stress [20]. An in vitro study showed that 1,8-cineole has a protective effect on oxidative stress induced by hydrogen peroxide (H_2_O_2_) in rat pheochromocytoma cells by enhancing the expression of CAT, SOD, and GPH-PX [8]. In addition, an in vivo study suggested that *Eucalyptus* essential oil containing with 67.85% 1,8-cineole improved the activity of SOD and decreased the content of MDA in the serum of Ross 308 broilers [16]. The present study was consistent with studies reporting that supplementation of 1,8-cineole enhanced antioxidant capacity and alleviated lipid peroxidation by increasing the activities of GSH-Px and SOD, TAOC, and decreasing the content of MDA in AA broilers. 1,8-cineole was reported to scavenge ROS in vitro [10]. Porres-martinez et al. [8] reported that 1,8-cineole enhanced the expression of SOD and GSH-PX and the ratio of nuclear vs. cytoplasmic Nrf2 and inhibited the production of intracellular ROS and the activity of caspase-3, which can protect rat pheochromocytoma cells from oxidative stress induced by hydrogen peroxide. Therefore, 1,8-cineole might inhibit the production of ROS, enhance the activity of antioxidant enzymes, and alleviate oxidative stress.

IgG, IgM, and IgA are the main immunoglobulins produced by activated B lymphocytes, which reflect the humoral immune status of the body [21]. The current study indicated that 1,8-cineole supplementation in the diet increased the serum levels of IgA, IgM, and IgG. To our knowledge, there is little literature reporting the effect of 1,8-cineole on serum immunoglobulin levels in broiler chickens. However, some studies have reported that plant essential oils containing 1,8-cineole have positive effects on the humoral immunity of broilers. Studies have shown that a blend of Eucalyptus and peppermint oils had a positive effect on the humoral immune response to Infectious bursal virus [22] and Newcastle disease virus in broiler chickens [23]. The specific mechanisms of the immunomodulatory effects of 1,8-cineole are still unclear. 1,8-cineole may induce the morphological and functional activation of mononuclear macrophages to stimulate phagocytosis of innate immune defense and thus improve the immune level of the body [24].

Under normal circumstances, the levels of pro-inflammatory factors and anti-inflammatory factors are balanced in healthy animals. Overexpression of cytokines such as TNF-α and IL-1β leads to an excessive inflammatory response [25]. Cytokines such as IL-4 and IL-10 play an anti-inflammatory role and inhibit macrophages from producing cytokines and other pro-inflammatory functions [26]. Studies in vitro and in vivo have shown that 1,8-cineole has anti-inflammatory effects. Jiang et al. [27] reported that 1,8-cineole alleviated LPS-induced vascular endothelial injury in mice by decreasing the level of IL-1β and increasing the level of IL-10. The protective effect of 1,8-cineole may be related to the up-regulation of PPAR-γ expression and down-regulation of P-NF-κB and p65 expression. 1,8-cineole also had a protective effect on LPS-induced injury of human umbilical vein endothelial cells by decreasing the gene expression of TNF-α and IL-1β mRNA [28]. The present study demonstrated that 1,8-cineole enhanced immune function and reduced the inflammatory response in broilers by decreasing TNF-α and IL-1β, levels and increasing IL-4 and IL-10 levels in serum in unchallenged broilers. This suggested that 1,8-cineole also has anti-inflammatory effects on broiler chickens, which may be achieved by inhibiting the associated inflammatory signaling pathways. Yadav et al. [7] demonstrated that 1,8-cineole inhibited the levels of TNF-α and IL-1β by selectively downregulating PRR pathways, including PRR receptors (TREM-1 and NLRP3) and common downstream signaling cascade partners (NF-κB, MAPK, MKP-1).

VH, CD, and VH/CD are commonly used to evaluate intestinal function. Longer villi provide more areas for absorption of nutrients, while deeper crypts indicate more stem cells for the renewal of intestinal epithelia [29]. Addition of myrtle (*Myrtus communis*) essential oil containing 18% 1,8-cineole improved intestinal morphology by increasing the VH and VH/CD of broiler chickens [30]. Dietary supplementation of sage (*Salvia officinalis* L.) extract containing 8.3% 1,8-cineole increased the jejunal villus length of hypertensive broilers chickens [31]. The present study showed that dietary supplementation of 1,8-cineole increased the VH and VH/CD in the jejunum and the ileum and CD in the ileum. Digestion and absorption of nutrients take place mainly in the small intestine. The possible reason for the decrease in the ratio of feed to gain in this study was related to the improvement in intestinal morphology, which led to the increase in nutrient absorption efficiency. 1,8-cineole improved the intestinal morphology of broilers, which might be related to its selective antibacterial activity. It has been proven that *Eucalyptus globulus* oil containing 83.65% 1,8-cineole has a strong inhibitory effect on *Enterococcus faecalis* and a slight inhibitory effect on *Lactobacillus rhamnosus* in vitro [32].

The intestinal mucosa is the first line of defense against bacteria and the adaptive humoral immune defense on its surface is mediated to a large extent by secreted IgA (SIgA) antibodies, which is the main humoral defense mechanism on the mucosal surface [33,34]. Studies have reported that adding a blend of thymol and eugenol to diets improves intestinal health by increasing SIgA levels in the small intestinal mucosa of broilers [35,36]. However, there are few studies about intestinal immunity of 1,8-cineole in broilers. Therefore, the results of our study showed that dietary supplementation of 1,8-cineole increased the content of SIgA in the intestinal mucosa of broilers. This suggested that 1,8-cineole can protect the villi from damage by stimulating the body’s humoral immunity to produce more antibodies on the intestinal mucosal surface [37].

## 5. Conclusions

In conclusion, dietary supplementation of 1,8-cineole improved the growth performance of broilers by improving antioxidant capacity, immunity, and intestinal morphology, and the optimal inclusion level of 1,8-cineole was found to be 20~30 mg/kg.

## Figures and Tables

**Table 1:** Ingredient composition and nutrient composition of basal diets (%, as-fed basis).

Ingredient (%)	Starter Phase (1–21 days)	Grower Phase (22–42 days)
Corn	60.13	61.53
Soybean meal	32.50	31.70
Fish meal	2.00	0.00
Soybean oil	1.50	3.00
Dicalcium phosphate	1.50	1.70
Limestone	1.34	1.15
DL-methionine (98%)	0.23	0.12
Sodium chloride	0.30	0.30
Premix ^1^	0.50	0.50
Total	100.00	100.00
Nutrient composition ^2^		
ME (MJ/kg)	12.59	13.22
Crude protein	20.57	19.53
Calcium	0.91	0.89
Total phosphorus	0.53	0.52
Lysine	1.12	1.06
Methionine + Cysteine	0.91	0.81

^1^ Premix provided per kg of diet: vitamin A, 9000 IU; vitamin D_3_, 3000 IU; vitamin E, 24 mg; vitamin K_3_, 1.8 mg; vitamin B_1_, 2.0 mg; riboflavin, 5 mg; vitamin B_6_, 3.0 mg; vitamin B_12_, 0.1 mg; nicotinic acid, 40 mg; calcium pantothenate, 15 mg; folic acid, 1 mg; biotin, 0.05 mg; Fe, 80 mg; Cu, 20 mg; Zn, 90 mg; Mn, 80 mg; iodine, 0.35 mg; Se, 0.3 mg. ^2^ ME was calculated values using NRC (1994) values; others were analyzed values.

**Table 2:** Effects of dietary 1,8-cineole supplementation on growth performance of broiler chickens.

Items ^1^	1,8-Cineole (mg/kg)					SEM ^2^	*p*-Value		
	0	10	20	30	40		ANOVA	Linear	Quadratic
Day 1–21									
ADG (g/d)	31.45	31.98	32.37	32.38	31.87	0.28	0.13	0.16	0.03
ADFI (g/d)	42.38	42.46	41.95	43.21	41.44	1.01	0.79	0.72	0.61
FCR (g/g)	1.35	1.33	1.30	1.33	1.30	0.03	0.79	0.40	0.79
Day 22–42									
ADG (g/d)	71.04 ^b^	71.37 ^b^	75.63 ^a^	75.78 ^a^	73.59 ^ab^	1.01	<0.01	0.01	0.02
ADFI (g/d)	135.15	134.20	138.32	138.15	138.19	1.33	0.10	0.03	0.65
FCR (g/g)	1.90 ^a^	1.88 ^ab^	1.83 ^b^	1.82 ^b^	1.88 ^ab^	0.02	0.04	0.14	0.01
Day 1–42									
ADG (g/d)	51.24 ^b^	51.67 ^b^	54.00 ^a^	54.08 ^a^	52.73 ^ab^	0.52	<0.01	<0.01	0.01
ADFI (g/d)	88.77	88.33	90.14	90.68	89.82	0.82	0.26	0.10	0.50
FCR (g/g)	1.73	1.71	1.67	1.68	1.70	0.01	0.04	0.07	0.01

^1.^ ADG = average daily gain; ADFI = average daily feed intake; FCR = ratio of feed to gain. ^2.^ SEM = total standard error of means (*n* = 6). ^a,b^ Means within a row with different superscripts are significantly different (*p* < 0.05).

**Table 3:** Effects of dietary 1,8-cineole supplementation on antioxidant capacity of broiler chickens in serum.

Items ^1^	1,8-Cineole (mg/kg)					SEM ^2^	*p*-Value		
	0	10	20	30	40		ANOVA	Linear	Quadratic
Day 21									
GSH-PX (U/mL)	202.82	231.55	243.66	238.03	229.58	14.73	0.36	0.21	0.11
T-SOD (U/mL)	138.86	128.05	130.42	141.76	142.90	9.78	0.75	0.49	0.38
TAOC (mmol/L)	0.37 ^b^	0.44 ^a^	0.44 ^a^	0.43 ^a^	0.42 ^a^	0.01	<0.01	0.06	<0.01
MDA (nmol/mL)	3.63	3.12	3.11	3.16	3.01	0.31	0.66	0.23	0.51
Day 42									
GSH-PX (U/mL)	306.76	355.77	335.21	343.38	355.94	15.68	0.19	0.10	0.46
T-SOD (U/mL)	140.53 ^b^	165.23 ^a^	178.13 ^a^	162.73 ^a^	165.40 ^a^	5.52	<0.01	0.01	<0.01
TAOC (mmol/L)	0.26 ^b^	0.36 ^a^	0.36 ^a^	0.38 ^a^	0.38 ^a^	0.02	<0.01	<0.01	0.01
MDA (nmol/mL)	3.88 ^a^	2.49 ^b^	2.20 ^b^	2.37 ^b^	2.34 ^b^	0.25	<0.01	<0.01	<0.01

^1.^ GSH-Px, glutathione peroxidase; SOD, superoxide dismutase; TAOC, total antioxidant capacity; MDA, malonaldehyde. ^2.^ SEM = total standard error of means (*n* = 6). ^a,b^ Means within a row with different superscripts are significantly different (*p* < 0.05).

**Table 4:** Effects of dietary 1,8-cineole supplementation on antioxidant capacity of broiler chickens in the liver.

Items ^1^	1,8-Cineole (mg/kg)					SEM ^2^	*p*-Value		
	0	10	20	30	40		ANOVA	Linear	Quadratic
Day 21									
GSH-PX (U/mg)	376.69	389.43	420.41	417.50	413.25	18.11	0.36	0.09	0.33
T-SOD (U/mg)	113.77	120.55	129.72	126.42	121.37	4.71	0.19	0.17	0.05
TAOC (mmol/g)	0.53	0.62	0.63	0.54	0.55	0.03	0.10	0.69	0.04
MDA (nmol/mg)	1.35 ^a^	1.15 ^b^	1.02 ^b^	0.99 ^b^	1.04 ^b^	0.06	<0.01	<0.01	0.01
Day 42									
GSH-PX (U/mg)	299.59	298.56	326.64	340.58	323.00	21.60	0.59	0.21	0.56
T-SOD (U/mg)	88.39 ^b^	100.70 ^ab^	104.43 ^a^	111.31 ^a^	107.58 ^a^	4.97	0.03	<0.01	0.13
TAOC (mmol/g)	0.45 ^b^	0.52 ^ab^	0.62 ^a^	0.62 ^a^	0.57 ^ab^	0.04	0.02	0.01	0.02
MDA (nmol/mg)	1.29 ^a^	1.06 ^b^	0.87 ^b^	0.97 ^b^	1.00 ^b^	0.07	<0.01	0.01	<0.01

^1.^ GSH-Px, glutathione peroxidase; SOD, superoxide dismutase; TAOC, total antioxidant capacity; MDA, malonaldehyde. ^2.^ SEM = total standard error of means (*n* = 6). ^a,b^ Means within a row with different superscripts are significantly different (*p* < 0.05).

**Table 5:** Effects of dietary 1,8-cineole inclusion on serum immunoglobulin levels of broiler chickens (g/L).

Items ^1^	1,8-Cineole (mg/kg)					SEM ^2^	*p*-Value		
	0	10	20	30	40		ANOVA	Linear	Quadratic
Day 21									
IgA	0.81 ^b^	1.05 ^ab^	1.24 ^a^	1.33 ^a^	1.06 ^ab^	0.09	<0.01	0.01	<0.01
IgG	6.21 ^d^	7.13 ^c^	7.74 ^bc^	8.76 ^a^	8.19 ^ab^	0.28	<0.01	<0.01	0.02
IgM	0.65 ^d^	0.80 ^c^	0.82 ^bc^	1.10 ^a^	0.94 ^b^	0.04	<0.01	<0.01	0.03
Day 42									
IgA	1.33	1.32	1.57	1.36	1.21	0.10	0.16	0.55	0.06
IgG	8.31	8.38	8.36	8.61	8.31	0.48	0.99	0.88	0.77
IgM	1.17	1.18	1.21	1.18	1.17	0.05	0.97	0.96	0.57

^1.^ IgG, immunoglobulin G; IgA, immunoglobulin A; IgM, immunoglobulin M.^2.^ SEM = total standard error of means (*n* = 6). ^a,b,c,d^ Means within a row with different superscripts are significantly different (*p* < 0.05).

**Table 6:** Effects of dietary 1,8-cineole inclusion on serum cytokines level of broiler chickens (pg/mL).

Items ^1^	1,8-Cineole (mg/kg)					SEM ^2^	*p*-Value		
	0	10	20	30	40		ANOVA	Linear	Quadratic
Day 21									
TNF-α	355.84	308.84	308.70	299.08	233.68	27.28	0.06	0.01	0.65
IL-1β	38.77	34.85	31.35	34.96	34.45	1.80	0.11	0.15	0.06
IL-4	8.45	8.96	11.09	10.60	9.66	0.69	0.07	0.08	0.04
IL-10	15.87 ^b^	16.53 ^b^	18.05 ^ab^	20.89 ^a^	15.72 ^b^	1.30	0.04	0.33	0.04
Day 42									
TNF-α	100.92 ^a^	93.28 ^ab^	94.16 ^ab^	87.39 ^b^	88.57 ^b^	3.12	0.04	<0.01	0.04
IL-4	4.12	4.48	4.58	4.20	4.11	0.34	0.80	0.79	0.28
IL-1β	30.90 ^a^	27.09 ^b^	26.02 ^b^	27.32 ^b^	27.55 ^b^	0.97	0.02	0.04	0.01
IL-10	12.14 ^b^	12.45 ^b^	13.65 ^a^	13.08 ^ab^	12.67 ^ab^	0.34	0.04	0.13	0.02

^1.^ TNF-α, tumor necrosis factor-α; IL-1β, interleukin-1β; IL-4, interleukin-4; IL-10, interleukin-10. ^2.^ SEM = total standard error of means (*n* = 6). ^a,b^ Means within a row with different superscripts are significantly different (*p* < 0.05).

**Table 7:** Effects of dietary 1,8-cineole supplementation on intestinal mucosa SIgA(ng/mg) content of broiler chickens.

Items	1,8-Cineole (mg/kg)					SEM ^1^	*p*-Value		
	0	10	20	30	40		ANOVA	Linear	Quadratic
Day 21									
Duodenum	251.14 ^b^	372.82 ^a^	371.47 ^a^	467.10 ^a^	450.70 ^a^	36.14	<0.01	<0.01	0.20
Jejunum	484.79 ^c^	512.93 ^c^	570.07 ^bc^	756.86 ^a^	631.98 ^b^	36.56	<0.01	<0.01	0.21
Ileum	501.56 ^b^	560.56 ^b^	637.71 ^ab^	624.37 ^ab^	737.68 ^a^	47.27	0.02	<0.01	0.92
Day 42									
Duodenum	302.76 ^b^	423.14 ^a^	468.26 ^a^	428.88 ^a^	387.13 ^ab^	34.26	0.03	0.12	<0.01
Jejunum	536.61	612.58	666.31	675.50	563.69	55.99	0.34	0.51	0.06
Ileum	311.82 ^b^	394.50 ^ab^	433.21 ^a^	486.82 ^a^	424.90 ^a^	33.74	0.02	0.01	0.04

^1.^ SEM = total standard error of means (*n* = 6). ^a,b,c^ Means within a row with different superscripts are significantly different (*p* < 0.05).

## Data Availability

The data presented in this study are available on request from the corresponding author.
